# Cariprazine as a transdiagnostic treatment for symptoms of depression across different disorders

**DOI:** 10.1192/j.eurpsy.2026.10611

**Published:** 2026-07-31

**Authors:** Z. B. Dombi, R. Csehi, Á. Barabássy

**Affiliations:** Global Medical Division, Gedeon Richter Plc, Budapest, Hungary

## Abstract

**Introduction:**

Symptoms of depression are prevalent across major depressive disorder (MDD), bipolar disorder (BD), and schizophrenia, yet treatment strategies are traditionally indication specific. Cariprazine, a dopamine D3/D2 partial agonist approved for all three disorders in the U.S., provides an opportunity to evaluate the efficacy of a single compound on depressive symptoms across diagnostic categories.

**Objectives:**

This meta-analysis aimed to pool clinical trial data to determine the efficacy of cariprazine versus placebo in alleviating depressive symptoms, regardless of primary diagnosis.

**Methods:**

Only randomized controlled trials comparing cariprazine with placebo were included. Data were drawn from post-hoc and pooled analyses across different psychiatric indications and dose ranges. The primary outcome was change from baseline in depressive symptom scores, standardized across trials by converting effect sizes to Cohen’s d.

Meta-analyses were performed in RStudio (version 2024.04.2+764) using the metafor package. A multilevel random-effects model accounted for overlapping placebo groups in trials with multiple dose arms, with comparisons grouped by study (random = ~1 | STUDY). Effect sizes are reported as pooled standardized mean differences (Cohen’s d) with 95% confidence intervals. Between-study heterogeneity was assessed using Q, I², and τ². Forest plots display individual and pooled estimates of cariprazine’s antidepressant effects.

**Results:**

Four trials in bipolar depression (MD-52, MD-53, MD-54, MD-56; doses 1.5–3 mg/day) were included, alongside five adjunctive MDD trials (MD-71, MD-72, MD-75, 301, 302; doses 1–4.5 mg/day) and three schizophrenia studies (MD-04, MD-05, MD-16; pooled dose range 1.5–9 mg/day). Across all indications, the pooled least square mean difference in depressive symptoms for cariprazine versus placebo was –1.45 (95% CI: –2.18 to –0.71), indicating a significant overall benefit. The largest effects were observed in bipolar depression and MDD, with smaller but positive effects in schizophrenia, where depressive symptoms were assessed via the PANSS Depression/Anxiety Marder factor score rather than dedicated depression scales.

**Image 1:**
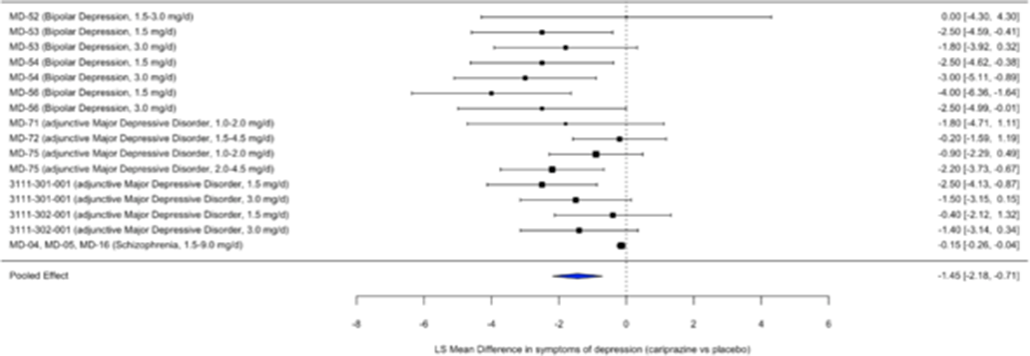
Image 1: Long description.

**Conclusions:**

Cariprazine significantly improved depressive symptoms across bipolar disorder, MDD, and schizophrenia. These findings support its potential as a transdiagnostic treatment for depression across psychiatric disorders.

**Disclosure of Interest:**

Z. Dombi Employee of: Gedeon Richter Plc, R. Csehi Employee of: Gedeon Richter Plc, Á. Barabássy Employee of: Gedeon Richter Plc.

## Long descriptions

### Image 1: Long description

The forest plot displays 16 individual study rows and a final pooled effect row. The X-axis at the bottom represents the L S Mean Difference in symptoms of depression, ranging from -8 to 6, with a vertical dashed line at 0 indicating no effect.

Individual studies are listed on the left with their condition and dosage. From top to bottom:

* M D-52 (Bipolar Depression, 1.5-3.0 mg/d): Mean 0.00 [-4.30, 4.30]

* M D-53 (Bipolar Depression, 1.5 mg/d): Mean -2.50 [-4.59, -0.41]

* M D-53 (Bipolar Depression, 3.0 mg/d): Mean -1.80 [-3.92, 0.32]

* M D-54 (Bipolar Depression, 1.5 mg/d): Mean -2.50 [-4.62, -0.38]

* M D-54 (Bipolar Depression, 3.0 mg/d): Mean -3.00 [-5.11, -0.89]

* M D-56 (Bipolar Depression, 1.5 mg/d): Mean -4.00 [-6.36, -1.64]

* M D-56 (Bipolar Depression, 3.0 mg/d): Mean -2.50 [-4.99, -0.01]

* M D-71 (adjunctive Major Depressive Disorder, 1.0-2.0 mg/d): Mean -1.80 [-4.71, 1.11]

* M D-72 (adjunctive Major Depressive Disorder, 1.5-4.5 mg/d): Mean -0.20 [-1.59, 1.19]

* M D-75 (adjunctive Major Depressive Disorder, 1.0-2.0 mg/d): Mean -0.90 [-2.29, 0.49]

* M D-75 (adjunctive Major Depressive Disorder, 2.0-4.5 mg/d): Mean -2.20 [-3.73, -0.67]

* 3111-301-001 (adjunctive Major Depressive Disorder, 1.5 mg/d): Mean -2.50 [-4.13, -0.87]

* 3111-301-001 (adjunctive Major Depressive Disorder, 3.0 mg/d): Mean -1.50 [-3.15, 0.15]

* 3111-302-001 (adjunctive Major Depressive Disorder, 1.5 mg/d): Mean -0.40 [-2.12, 1.32]

* 3111-302-001 (adjunctive Major Depressive Disorder, 3.0 mg/d): Mean -1.40 [-3.14, 0.34]

* M D-04, M D-05, M D-16 (Schizophrenia, 1.5-9.0 mg/d): Mean -0.15 [-0.26, -0.04]

The final row, Pooled Effect, is represented by a blue diamond centered at -1.45 with a 95% confidence interval of [-2.18, -0.71]. Most studies show a trend favoring cariprazine, as indicated by data points and error bars positioned to the left of the zero line.

Navigate back to Image 1:.

